# Chitosan nanoparticles and green synthesized silver nanoparticles as novel alternatives to antibiotics for preventing *A.* *hydrophila subsp. hydrophila* infection in Nile tilapia, *Oreochromis niloticus*

**DOI:** 10.1080/23144599.2023.2205338

**Published:** 2023-05-08

**Authors:** Salah M. Aly, Alaa Eldin Eissa, Nashwa Abdel-Razek, Asmaa O. El-Ramlawy

**Affiliations:** a Department of Pathology, Faculty of Veterinary Medicine, Suez Canal University, Ismailia, Egypt; b Department of Aquatic Animal Medicine & Management, Faculty of Veterinary Medicine, Cairo University, Giza, Egypt; c Department of Fish Health and Management, central laboratory for Aquaculture Research, Agriculture Research center, Sharqia, Egypt; d Department of Aquaculture Diseases Control, Fish Farming and Technology Institute, Suez Canal University, Ismailia, Egypt

**Keywords:** Chitosan, Silver, Nanoparticles, *Aeromonas hydrophila* subsp. *hydrophila*, MDR, Tilapia

## Abstract

Recently, nanoparticles have attracted attention as a preventive tool for certain infectious diseases affecting fish in aquaculture. Furthermore, freshwater fishes are frequently vulnerable to summer mass morality caused by Aeromonas bacteria. In this regard, we focused on the evaluation of the in vitro and in vivo antimicrobial activity of chitosan (CNPs) and silver (AgNPs) nanoparticles against *Aeromonas hydrophila subsp. hydrophila*. CNPs and AgNPs were prepared at a mean particle size of 9.03 and 12.8 nm and a charge equalled+36.4 and −19.3 mV for CNPs and AgNPs, respectively. *A.* *hydrophila subsp. hydrophila, Aeromonas caviae, and Aeromonas punctata* were retrieved and identified by traditional and molecular techniques. The sensitivity of the obtained bacteria to eight different antibiotic discs was also tested. The antibiotic sensitivity studies revealed the presence of multidrug-resistant (MDR) *Aeromonas* species (spp.). The bacterium that showed the highest multidrug resistance against the tested antibiotic discs was *Aeromonas hydrophila subsp. hydrophila*. Therefore, CNPs and AgNPs were in vitro tested against the isolated bacterium and exhibited inhibition zones of 15 and 25 mm, respectively. TEM images also showed that CNPs and AgNPs had an antagonistic action against the same bacterium causing loss of architecture and bacterial death.

## 1. Introduction

Nanotechnology involves dealing with materials with unique features at nanoscale dimensions (1–100 nm) that enable novel applications such as vaccines, diagnostics, pharmaceuticals, etc [1]. In agriculture and aquaculture, nanotechnology demonstrates a wide range of interdisciplinary activities. It can potentially change the aquaculture and fisheries industries by providing new tools for rapid disease diagnosis and improving fish’s ability to absorb nutrients [2].

Although tilapia fish can withstand harsh environmental conditions, bacterial disease outbreaks remain one of the major limiting factors that affect tilapia production in intensive cultures [3].

Due to the widespread mortalities brought on by Motile Aeromonas Septicemia (MAS), *Aeromonas hydrophila* is the most common Gram-negative waterborne bacteria and is responsible for global economic losses in aquaculture. The antimicrobial efficacy against this pathogen was limited due to its unique capacity to generate biofilms. It also poses a risk to workers and farmers who come into contact with infected fish, as it causes gastroenteritis and several systemic disorders in both immunocompetent and immunocompromised individuals [4,5].

To control bacterial infections in aquaculture, antibiotics are routinely used. Antibiotics corrupt the ecosystem and do not differentiate but kill both normal inhabitant flora and pathogenic bacteria of the treated host. Moreover, their abuse has caused bacteria to create antibiotic-resistant strains [6]. Antibiotic overuse in aquaculture increases multidrug-resistant *A. hydrophila* strains, making antimicrobial resistance monitoring in aquaculture is essential [7]. Due to this, selecting the best antibacterial medications to treat and prevent bacterial septicaemia in fish farms can be challenging for veterinarians [8].

Recently, the use of nanoparticles as antimicrobial additives for preventing bacterial, fungal, and viral illnesses in aquaculture has gained attention, with the issue of antibiotic-resistant bacteria receiving particular attention [9].

Because of their unique characteristics, chitosan nanoparticles (CNPs) demonstrated a significant antimicrobial activity against Gram-positive and Gram-negative bacteria, and other fungi [10]. Chitosan is a biocompatible cationic polysaccharide and considered the deacetylated product of chitin, the natural polymer found abundantly in the shells of crustaceans, insects, and in fungi. It is used to create a wide range of nanocomposites with improved antibacterial and immunostimulatory properties [11,12]. CNPs are one of the most promising biodegradable natural nano-products for aquaculture disease prevention and therapy [13]. The use of CNPs as a superior antibacterial agent has gained popularity due to the rapid advancement of green chemistry [14].

**Figure 1.**
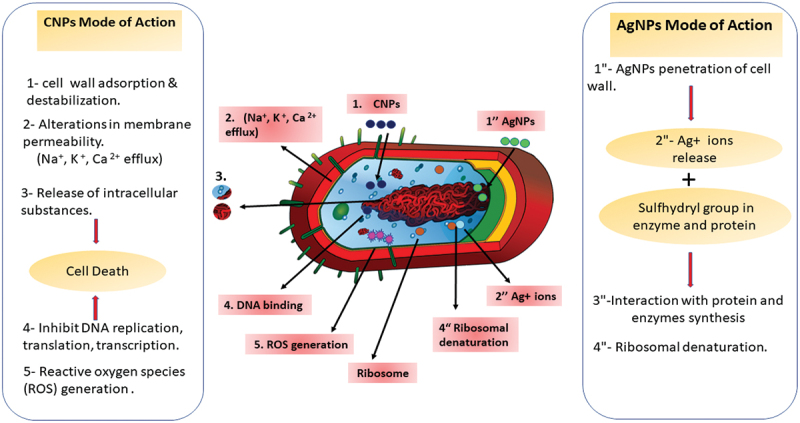
Simplified graph of the possible mechanisms of action of CNPs and AgNPs against fish bacterial pathogens.

AgNPs are considered promising antibacterial agents. In aquaculture and agriculture, AgNPs are still relatively new, despite the research on these nanoparticles as an antibacterial has been expanding recently. There are *in vitro* results; however, the *in vivo* data are inadequate [15]. The mechanisms of antibacterial activity of AgNPs and CNPs were illustrated in (Figure 1).

The goal of the current study was to assess the *in vitro* and *in vivo* antibacterial activity of synthetized CNPs and AgNPs against *A. hydrophila* subsp. *hydrophila* in Nile tilapia culture.

## 2. Materials and methods

### 2.1. Ethical statement

The experimental design was approved by the Research Ethical Committee of the Faculty of Veterinary medicine, Suez Canal University, Ismailia, Egypt (Ethical approval No. 2020005).

### 2.2. Preparation of nanoparticles

#### 2.2.1. Preparation of chitosan nanoparticles (CNPs)

Chitosan powder was purchased from HiMedia Chemicals, India, CAS No. 9012-76-4. The molecular weight of the purchased particles ranged between 3800 and 20,000 daltons, and their de-acetylation degree (DD) was about 75%. Chitosan powder was originated from chitin, the natural chitinous shell of crustaceans (shrimps, crabs, and crays). CNPs were prepared based on ionic gelation mechanism of chitosan with tripolyphosphate (TPP) anion. The synthesis process was performed at the Animal Health Research Institute (AHRI), Dokki, Giza, Egypt, according to **Zhang et al**. [16]. Briefly, chitosan was dissolved in 1% acetic acid and agitated for 1 h to make the solution clear. Sodium tripolyphosphate (0.5 mg/ml) was dissolved in deionized water. Under magnetic stirring at room temperature, 1.0 ml of TPP was added dropwise to 100 ml of chitosan solution. The mixture was stirred for 20 min, followed by sonication. The resulting suspension was subsequently centrifuged at 10,000 rpm for a further 20 min. Supernatants were discarded, and the precipitate was suspended in distilled water and then freeze-dried before further use or analysis. The freeze-dried CNPs were suspended in deionized distilled water for characterization or directly used for further experiments and stored at 4 degree Celsius (°C).

#### 2.2.2. Preparation of silver nanoparticles (AgNps)

Gum arabic (GA), *Acacia arabica*, a natural sticky exudate extracted from Acacia tree stems and branches was known to have powerful reducing properties. Dried powder of gum arabic was purchased from a local market in Ismailia, Egypt, and used as a reducing agent for silver nitrate (AgNO3) to synthesize AgNPs. AgNO_3_ crystals were purchased from Sigma-Aldrich, Massachusetts, USA. The method of preparation was fully described by **Aly et al**. [17].

### 2.3. Characterization of CNPs and AgNPs

The obtained solutions were sent to Nawah Scientific lab, Egypt, to analyse the physical characteristics of CNPs and AgNPs. Using a Zeta sizer 3000 HSA and dynamic light scattering (DLS) analysis, the average particle size and zeta potential (surface charge) were determined (Malvern Instruments Ltd, UK). Using TEM (Jeol Jem-2100, Tokyo, Japan), the morphology of the prepared particles was studied as described by **Liu et al** [18].

### 2.4. Bacterial isolation and identification

#### 2.4.1. Sample collection and study areas

A total of 110 diseased Nile tilapia of both sexes (60 ± 5 gm), exhibiting clear clinical signs of septicaemia as defined according to **Austin and Austin** [19], were gathered from fish farms in Ismailia Governorate, Egypt (30° 36’ 15.37“N. 32° 16’ 20.10” E), throughout the four seasons of 2021. Freshly killed fish were transported to the bacteriology lab in an icebox at Fish Farming and Technology Institute in Ismailia, Egypt. Alive ones were placed in a labelled plastic bag with water (approximately one-fourth of the total bag capacity) for dissection and bacterial isolation. Prior to dissection, fish were anaesthetized by immersion in a 40 mg/L solution of tricaine methane sulphonate (MS-222) [20].

#### 2.4.2. Isolation of pathogenic bacteria

The post-mortem (PM) examination was carried out after disinfecting the external surface of each tilapia fish with ethyl alcohol (70%). A loop of liver, kidney, and spleen was enriched in sterile tubes with tryptic soy broth (TSB, Oxoid®, USA) and incubated for 18–24 h at 37°C. Each incubated tube had a loopful streaked on tryptic soy agar (TSA, Oxoid®, USA) and incubated at 37°C for 18–24 h. Subcultures were kept on TSA slopes at refrigerator till identification [21]. The identified isolates were kept at − 25°C in TSB with 20% (v/v) glycerol.

#### 2.4.3. Biochemical identification of the isolated bacteria

Standard biochemical tests (such as the oxidase and catalase tests), colony morphology, Gram staining, and cell arrangement description were used to identify three clinical isolates as described by **Tarrand and Gröschel** [22]. Using API20E strip system (Bio-Merieux, France), the biochemical analysis was performed according to the manufacturer’s instructions.

#### 2.4.4. Molecular identification and phylogenetic analysis

The highly conserved 16S rRNA gene was used to confirm the presumedly identified three representative isolates at Animal Health Research Institute (AHRI), Dokki, Giza Governorate [23]. According to the manufacturer’s instructions, genomic DNA was extracted from overnight cultures on tryptone soy agar (TSA) (Oxoid®, USA) using the QIAamp DNA Mini Kit (QIAGEN GmbH, Hilden, Germany), Cat. No. 51304. In a T3 Thermal cycler **(Biometra, Germany)**, the universal primer; forward: (5-AGAGTTTGATCMTGGCTCAG-3), and reverse: (5-TACGGYTACCTTGTTACGACTT-3) (**Metabion, Germany)** were used to amplify the 16S rRNA gene according to **Lagacé et al**. [24]. Agar gel electrophoresis images of the amplified DNA were also captured. The unassembled raw sequences from our investigation were put together and compared to the other sequences in GenBank using BLASTN. The retrieved accession numbers were used to identify the nucleotide sequences that were deposited in GenBank. A phylogenetic tree was created via the neighbour-joining method using MEGA X.11 software to determine the genetic distance. Bootstrap analysis was used to test the level of confidence for each branch at 1,000 repetitions [25].

### 2.5. Antimicrobial sensitivity testing

The susceptibility of the identified bacteria to eight widely used aquaculture antimicrobial agents was tested using the disc diffusion technique. The antimicrobials tested (Oxoid, England, UK) were amoxycillin (AML, 25 µg), streptomycin (S, 10 µg), ciprofloxacin (CIP, 5 µg), ceftriaxone (CRO, 30 µg), enrofloxacin (ENR, 5 µg), oxytetracycline (OT, 30 µg), trimethoprim/sulfamethoxazole (SXT, 25 µg), and amoxycillin clavulanic acid (AMC, 30 µg). The disc diffusion assay was carried out according to the guidelines proposed by the Clinical and Laboratory Standards Institute (CLSI) [26].

### 2.6. *Pathogenicity test (Koch’s postulate) for isolated* A. hydrophila *subsp*. hydrophila

To obtain the median lethal dose (LC_50_) of *A. hydrophila* subsp. *hydrophila*, the most frequent strain in this study with a higher resistance rate to different antibiotics, the pathogenicity test (the acute mortality test) was conducted. A single colony of the bacterium was added to TSB and incubated at 37°C for an entire night. The culture medium was thoroughly cleaned twice with phosphate buffer saline (PBS), then centrifuged at 3000 rpm for 10 min. The bacterial suspension was adjusted using a spectrophotometer (acculab Vs-85, USA) at optical density (OD_600_) into five concentrations (1.5 × 10^8^, 1.5 × 10^7^, 1.5 × 10^6^, 1.5 × 10^5^, 1.5 × 10^4^ CFU/mL) [27].

Fish Farming and Technology Institute rearing unit in Ismailia, Egypt, provided 180 healthy Nile tilapia fish of both sexes (60 ± 5.2 gm), stocked in 18 (200-L) fibreglass tanks with 10 fish per tank with constant aeration and flow of dechlorinated water. Various water parameters, including temperature, pH, and dissolved oxygen were measured daily, the fish were acclimated for two weeks and fed a commercially balanced diet. Three replicates of the tested fish were distributed evenly and randomly among six groups (30 fish per group). *A. hydrophila* subsp. *hydrophila* was introduced into five of these groups at various concentrations, while physiological saline was inoculated at the control group. All fish were anaesthetized in benzocaine solution (100 mg/mL) before inoculation and the five challenge groups were each intraperitoneally injected with 1 mL/kg of 1.5 × 10^8^, 1.5 × 10^7^, 1.5 × 10^6^, 1.5 × 10^5^, and 1.5 × 10^4^ CFU/mL, respectively, of *A. hydrophila* subsp. *hydrophila* according to **Vaneci-Silva et al**. [28]. The dose was modified to 0.3 mL per fish, this dose induced the clinical signs of the injected pathogen and different survival percentages in the inoculated groups [10]. Daily records of the fish mortalities and clinical signs were kept for 10 days. By re-isolating, the injected bacteria from the moribund fish, the cause of death was confirmed. The LC_50_ values were estimated according to **Miller and Tainter** [29]. The experiment was conducted by the guidelines approved by the Research Ethics Committee at the Faculty of Veterinary Medicine, Suez Canal University, Egypt.

### 2.7. In vitro *antibacterial activity of CNPs and AgNPs* against A. hydrophila *subsp*. hydrophila *using disc diffusion method*

Disposable Petri dishes were filled with a sterilized TSA medium. After solidification, the plates were inoculated in triplicate with 0.1 mL bacterial suspension at 1.0 × 10^8^ CFU/mL [30]. Some sterilized paper discs (6 mm in diameter) were pipetted with 50 µL of CNPs and others were pipetted with 50 µL of AgNPs and allowed to dry in an incubator. Once the discs were dry, they were applied to the agar surface and incubated at 30°C for 24 h. The diameter (mm) of inhibition zones caused by CNPs and AgNPs immersed discs was measured according to **Liu et al** [18].

### 2.8. *Antimicrobial activity of CNPs and AgNPs against A.* *hydrophila subsp*. *hydrophila* using TEM

*A.* *hydrophila subsp. hydrophila* was sub-cultured with 20 μg/mL of CNPs and AgNPs in separate falcon tubes containing tryptic soy broth. Twenty-four hours of incubation at 30°C was followed by 15 min of centrifugation (3000 rpm) to remove the supernatants. The precipitate was collected for examination via TEM (JEOL JEM-2100, Japan) at the EM unit, Mansoura University, Egypt, according to **Kinner et al** [31].

### 2.9. In vivo *studies on the prepared nanoparticles*

#### 2.9.1. Design of feeding experiment

One hundred and eighty (180) Nile tilapia of both sexes (101.5 ± 0.5 g) were kept in an indoor aerated fibreglass tank (1 m^3^ capacity) and fed on a basal diet for two weeks to acclimate Nile tilapia to indoor conditions. After that, fish were randomized into three equal groups in triplicate (60 fish/group) in 9 fibreglass tanks (200 cm × 200 cm, 20 fish/each). Using tank’s air pump, each fish tank was supplied with a compressed air via air stones. The first fish group (control) was only fed a commercial diet (30% crude protein) from Skretting Co., Egypt, while the second group was fed a commercial diet mixed with CNPs (2.0 g/kg diet). This dose was chosen as referred by **Abdel-Tawwab et al**. [32] and was tolerable to Nile tilapia, and the third group was fed the commercial diet mixed with AgNPs (1.0 mg/kg diet). The chosen dose of AgNPs had a protective non-toxic effect on tilapia fish [33,34]. To prepare the diets, the CNPs and AgNPs were suspended separately in 100 ml per kg of ground commercial diet and blended for 30 min to make a paste of each diet. The pastes were separately passed through a grinder and pelleted through a 1-mm diameter paste extruder. The diets were oven dried at 55°C for 24 h. Fish in all groups were fed 3% of their body weight twice daily (9:00 a.m. and 2:00 p.m.) for 60 days.

#### 2.9.2. Sample size calculation

A total sample size of 30 fish to conduct the challenge experiment were sufficient to detect an effect size of 0.22 at a power of 0.95 (95%) and partial eta squared of 0.05. Sample size was calculated using G*power application version 3.9.6 [35].

#### 2.9.3. Challenge experiment

After two months (end of the experiment), 30 fish from the three treated groups were collected and divided into three tanks (10 fish from each group per tank). Fish groups were intraperitoneally injected with a suspension of pathogenic bacteria (1 mL/kg of 3.0 × 10^7^ CFU/mL of 24 h live *A. hydrophila* subsp. *hydrophila*). The bacterial concentration was determined by a spectrophotometer at OD_600_ [36]. Daily counts of dead fish were recorded to plot the survival analysis using GraphPad prism version 8.0.2.263. The relative level of protection (RLP) of the challenged fish was calculated using the equation of **Ruangpan et al**. [37].

## 3. Results

### 3.1. Characterization of prepared nanoparticles

#### 3.1.1. Characterization of CNPs

**Figure 2.**
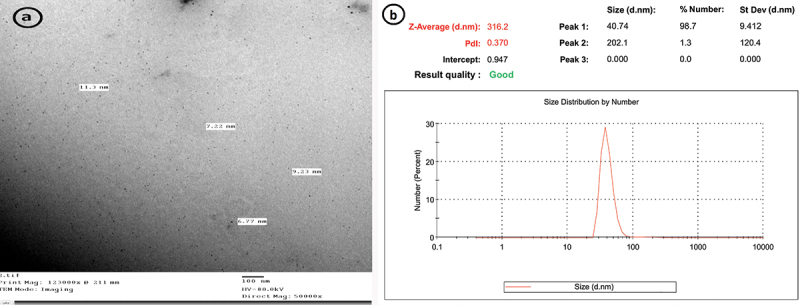
Morphological characteristics of prepared CNPs, a: TEM image showed the individual particle size ranges from 6.77 to 11.3 nm, b: the average particle size determined by DLS (316.2) nm.

**Figure 3.**
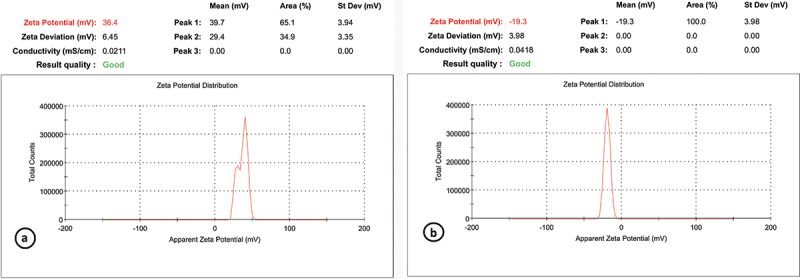
Zeta potential of CNPs and AgNPs; (a): revealed positively charged CNPs with a mean charge of +36.4 mV, (b): AgNPs exhibited a mean charge of (−19.3) mV.

The CNPs size distribution profile represented a typical nanoparticle batch with a diameter range of 6.77–11.3 nm and mean size of 9.03 nm (Figure 2a). DLS analysis was also used to determine the average particle size, which was approximately 312.6 nm in diameter, as shown in (Figure 2b). The TEM was utilized to examine the morphological properties of CNPs. The image of individual chitosan-TPP nanoparticles revealed a collection of spherical particles with distinct boundaries. The zeta potential (surface charge) was approximately+36.4 mV (Figure 3a).

#### 3.1.2. Characterization of AgNPs

**Figure 4.**
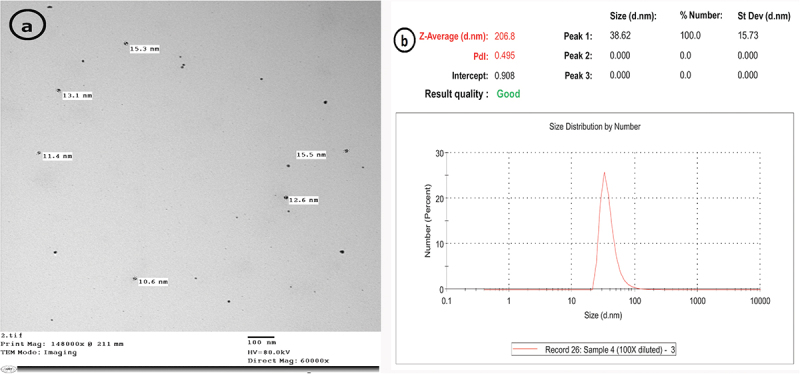
Morphological characteristics of AgNPs (a): TEM image showed; size ranges between 10.6 and 15 nm; (b): DLS revealed an average particle size of 206.8 nm.

AgNPs size profile represented identical nanoparticles with a range diameter of 10.6–15 nm and a mean size of 12.8 nm. The surface charge of silver nanoparticles was about (−19.3 mV) (Figure 3b). The morphological characteristics were examined using TEM (Figure 4a); the particles showed clear round boundaries. The average particle size of AgNPs measured by DLS was (206.8) nm (Figure 4b).

### 3.2. Bacterial strains isolation and identification

#### 3.2.1. Clinical and post-mortem examination of diseased fish

**Figure 5.**
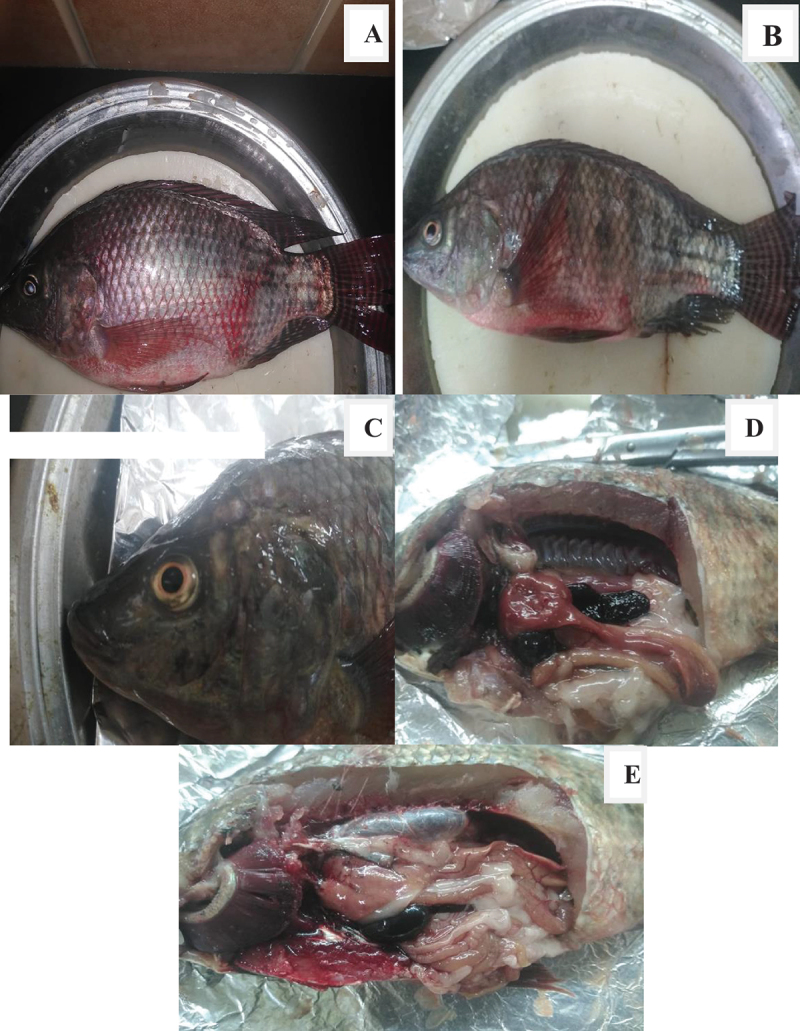
External and internal findings of diseased Nile tilapia fish.

The external examination of diseased tilapia fish revealed pale body surface, rot of the fins, exophthalmia, eyes with a cloudy appearance, abdominal distension, and haemorrhagic patches on the skin and abdomen. The darkened dorsal part of the body and mild hyperaemia on the pectoral and ventral fin bases were also observed. Macroscopic findings revealed the presence of pale gills (Figure 5A-C) .

A- Abdominal distension and hemorrhage. B- Hyperaemia at the base of the fins.

C- Cloudy, hemorrhagic eye. D- Enlarged and congested spleen.

E- Enlarged gall bladder, abdominal serous fluid accumulation.

The internal necropsy findings of diseased tilapia fish exhibited severe haemorrhagic spots on the liver, serous fluid accumulation in the intestine and abdominal cavity, liver congestion, enlarged congested spleen, and an emerald-black secretion filled the gall bladder (Figure 5D-E) .

#### 3.2.2. Isolation of pathogenic bacteria

Eighty isolates were retrieved from 110 moribund Nile tilapia fish and identified using routine morphological and bacteriological techniques. Three representative ones were subjected to a molecular biology lab for a definite diagnosis. The total number and percentage of the isolated bacteria during the four seasons of the study period were illustrated in Table (1). The frequent distribution of the recovered bacteria isolated from the liver, kidney, and spleen was reported in Table (2).

**Table 1.** The identified bacterial isolates which were obtained from the examined Nile tilapia fish during different seasons of the study period.

Isolated bacteria	Identified isolates	Number and percentages of retrieved isolates				
		Winter	Spring	Summer	Autumn	Total
*Aeromonas* spp.	*A. hydrophila* subsp. *hydrophila*	2 (28.60%)	10 (43.50%)	18 (54.50%)	4 (23.50%)	34 (42.50%)
	*A. caviae*	4 (57.20%)	7 (30.50%)	10 (30.30%)	10 (58.80%)	31 (38.75%)
	*A. punctata*	1 (14.20%)	6 (26%)	5 (15.20%)	3 (17.70%)	15 (18.75%)
Total	No.	7	23	33	17	80
	%	8.75	28.75	41.25	21.25	100

**Table 2.** Frequency of bacterial isolates from liver, kidney, and spleen of moribund Nile tilapia.

Isolated bacteria	Identified isolates	Number and percentages of retrieved isolates			
		Liver	Kidney	spleen	Total
*Aeromonas* spp.	*hydrophila* subsp. *hydrophila*	9 (81.80%)	15 (37.50%)	10 (34.50%)	34 (42.50%)
	*caviae*	0	16 (40%)	15 (51.70%)	31 (38.75%)
	*punctata*	2 (18.20%)	9 (22.50%)	4 (13.8%)	15 (18.75%)
Total	No.	11	40	29	80
	%	13.75	50	36.25	100

#### 3.2.3. Biochemical identification of the retrieved isolates

The isolates were subjected to primary conventional tests, including oxidase test and catalase test, and haemolysis ability by culturing on blood agar media. The isolates were positive for oxidase test and catalase test, β-haemolytic, and glucose fermenters. Analytical Profile Index (API20E) strip system was utilized to identify the three retrieved isolates. The code numbers on API20E strips were 5,265,124, 7367125, and 7,765,777, respectively.

#### 3.2.4. Molecular identification of isolated bacteria

**Figure 6.**
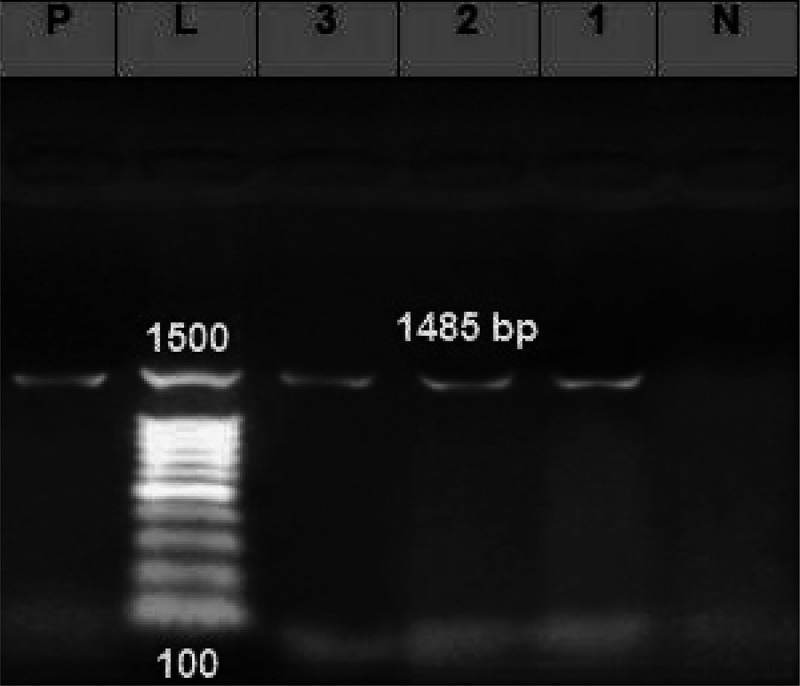
Gel electrophoresis image of the isolated bacteria’s PCR product (*n* = 3), L: a molecular weight ladder; p: a positive control; and N: a negative control; and (1), (2), and (3) are the DNA products of the bacterial isolates that have been amplified to a length of about 1485 base pairs (bp).

**Figure 7.**
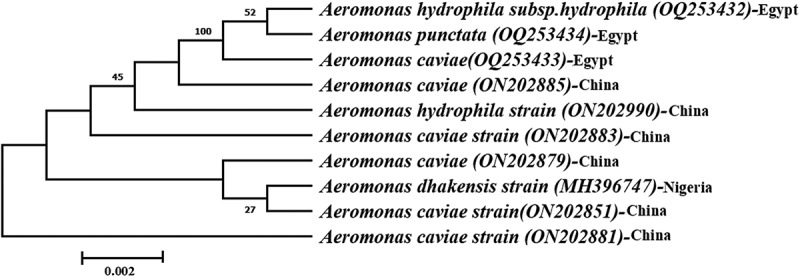
The phylogenetic tree of the identified Aeromonas spp. The Unrooted tree was generated using the Molecular Evolutionary Genetic Analysis (MEGA X 11.0) software via the neighbor-joining method based on 16S rRNA nucleotide sequences.

The PCR products of the three strains were subjected to gel electrophoresis and 16S rRNA sequencing. The PCR revealed the presence of non-specific amplification products for *Aeromonas* strains at 1485 bp (Figure 6). The assembled 16S rRNA gene sequences of the three strains were submitted to GenBank databases under the accession numbers of OQ253432, OQ253433, and OQ253434, respectively. The strains were identified as *A. hydrophila* subsp. *hydrophila, A. caviae*, and *A. punctata*. The neighbour-joining phylogenetic tree was constructed based on the sequenced 16S rRNA genes of the identified *Aeromonas* spp. The phylogenetic tree of the ITS regions of *Aeromonas* spp. revealed two main clusters (Figure 7). The first cluster showed that *Aeromonas* sequences retrieved in this study were grouped with sequences from China.

### 3.3. In vitro *antimicrobial sensitivity testing of isolated pathogens*

In this study, all the tested Aeromonads were ciprofloxacin, ceftriaxone, streptomycin, and enrofloxacin sensitive. Among *A. hydrophila* subsp. *hydrophila*, we found that the resistance rate was significantly higher for amoxycillin – clavulanic acid (AMC) (94.2%, 32/34) and amoxycillin (AML) (88.2%, 30/34); respectively. Among *A. caviae*, amoxycillin (AML) (93.5%, 29/31) and amoxycillin – clavulanic acid (AMC) (90.3%, 2831/) showed higher percentages of resistance. The highest level of resistance for *A. punctata* was for oxytetracycline (Table 3).

**Table 3.** The antibiogram of the three retrieved Aeromonas spp.

Antibiotic discs		*A. hydrophila* subsp. *hydrophila*		*A. caviae*		*A. punctata*		
		No.	%	No.	%	No.	%	
Ciprofloxacin (CIP)	S I R	34 0 0	100 0 0	31 0 0	100 0 0	15 0 0		100 0 0
Ceftriaxone (CRO)	S I R	34 0 0	100 0 0	31 0 0	100 0 0	15 0 0		100 0 0
Amoxicillin–clavulanic acid (AMC)	S I R	0 2 32	0 5.8 94.2	0 3 28	0 9.7 90.3	0 0 15		0 0 100
Amoxicillin (AML)	S I R	0 4 30	0 11.8 88.2	0 2 29	0 6.5 93.5	0 0 15		0 0 100
Oxytetracycline (OT)	S I R	10 9 15	29.4 26.5 44.1	5 10 16	16.1 32.2 51.7	3 4 8		20 26.7 53.3
Trimethoprim–sulfamethoxazole (SXT)	S I R	24 0 10	70.6 0 29.4	25 6 0	80.6 19.4 0	15 0 0		100 0 0
Streptomycin (S)	S I R	24 10 0	70.6 29.4 0	15 16 0	48.4 51.6 0	15 0 0		100 0 0
Enrofloxacin (ENR)	S I R	34 0 0	100 0 0	15 16 0	48.4 51.6 0	15 0 0		100 0 0

### 3.4. Pathogenicity test of *A.* *hydrophila subsp*. *hydrophila*

**Figure 8.**
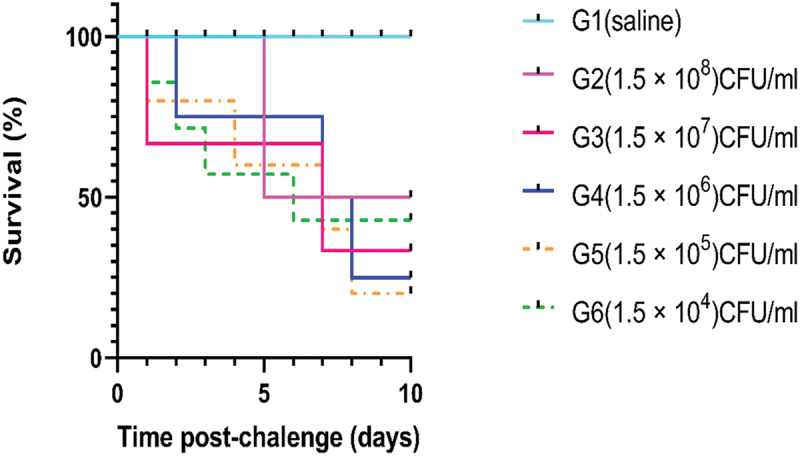
Graph-pad prism survival plot of Nile tilapia showed the pathogenicity.

**Figure 9.**
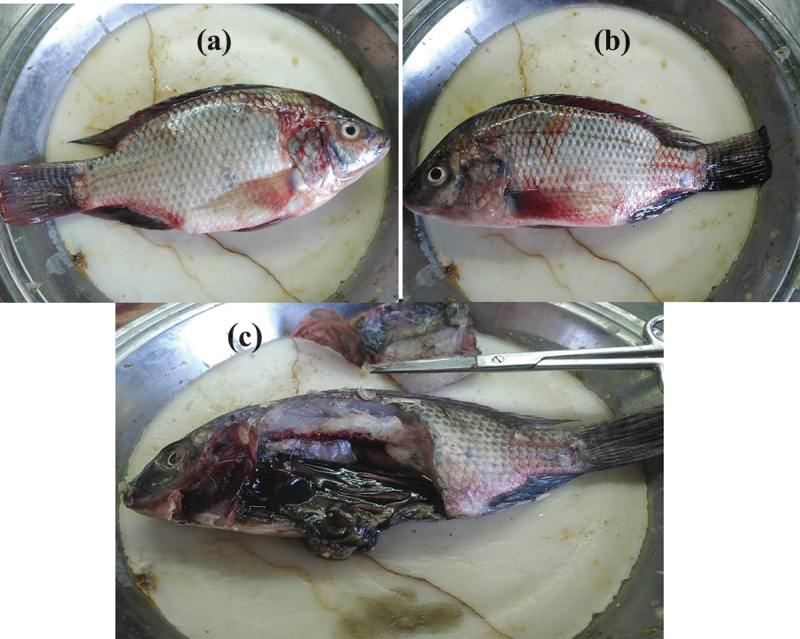
External and internal findings of Nile tilapia after conducting the pathogenicity test showing (a), (b): hemorrhagic patches on the fish body surface, hyperemia of fins; (c): abdominal cavity filled with exudates, congested internal organs, enlarged gall bladder filled with emerald-black secretion, and empty intestine.

LC_50_ of *A. hydrophila* subsp. *hydrophila* to Nile tilapia was determined as 1.5 × 10^5^ CFU/mL. Significantly fewer fish number was survived in the group administered the highest dose (1.5 × 10^8^ CFU/mL). The group exposed to the lowest dose (1.5 × 10^4^ CFU/mL) had a higher percentage of survivors (Figure 8). The bacteria-inoculated fish displayed the same clinical signs as naturally diseased fish, demonstrated the recovered strain’s virulence. The clinical signs were weakness, loss of appetite, slower movement, swimming closer to the surface, hyperaemia of fins, and red patches in gut regions. The clinical signs became noticeable 12 h after infection and persisted for 96 h. The first clinical signs of disease, including changes in feeding and swimming behaviour, haemorrhagic patches on the surface, and fin hyperaemia, appeared 14 h after the challenge (Figure 9a-b). The abdominal cavity filled with exudates, congested internal organs, an enlarged gall bladder filled with emerald-black secretion, and an empty intestine filled with gas bubbles (Figure 9c). These clinical signs were observed 7 h after the challenge. Mortality began 24 h after the challenge in all challenged groups. There was no mortality or abnormal behaviour in the control fish group.

### 3.5. In vitro antibacterial activity of CNPs and AgNPs against *A.* *hydrophila subsp. hydrophila*

CNPs and AgNPs exhibited *in vitro* inhibitory activities against *A. hydrophila* subsp. *hydrophila*. The inhibition zones were 15 and 25 mm in diameter, respectively, of A. hydrophila subsp. hydrophila. Different curves represent the percentage of fish survival (*n* = 30 per group), (G1): control group; (G2,3,4,5,6): fish injected with 1.5 × 10^8^, 1.5 × 10^7^, 1.5 × 10^6^, 1.5 × 10^5^, and 1.5 × 10^4^ CFU/mL, respectively.

### 3.6. Antibacterial action of CNPs and AgNPs against *A.* *hydrophila subsp. hydrophila* using TEM

#### 3.6.1. CNPs’ antibacterial activity via TEM

**Figure 10.**
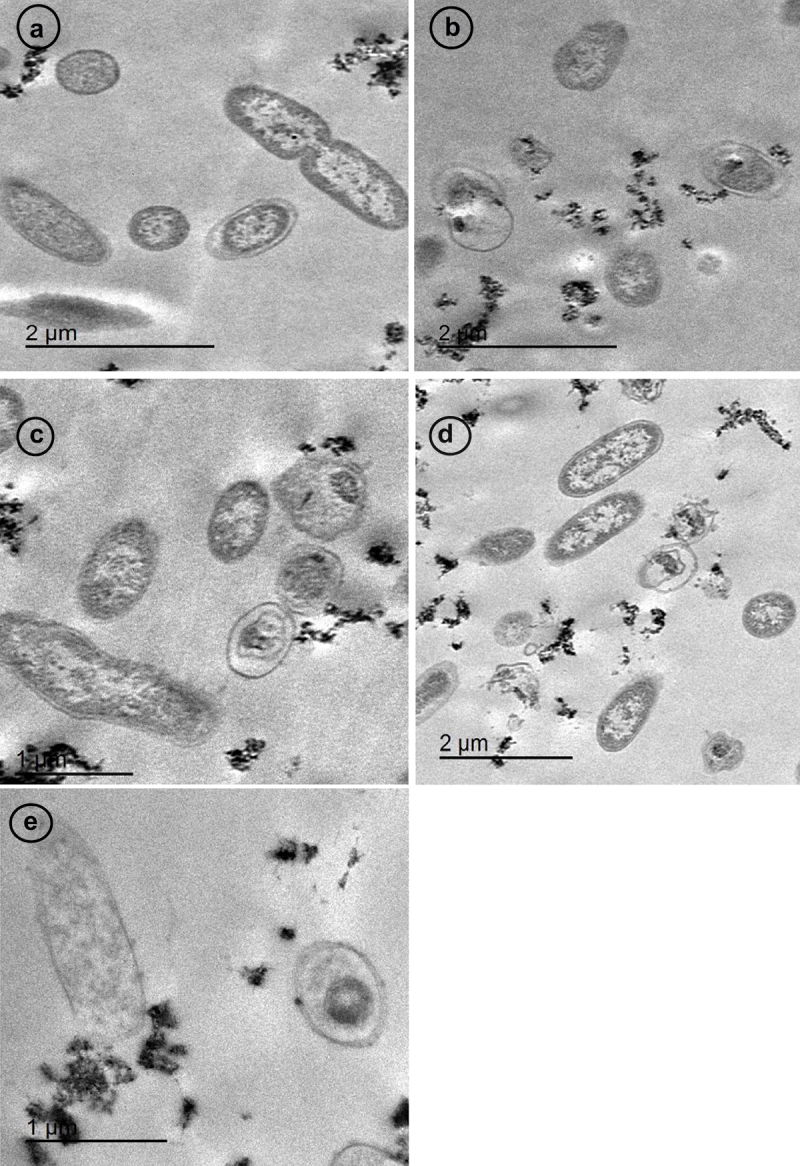
TEM images showed the microscopic alterations caused by CNPs against *A.* *hydrophila subsp. hydrophila*; a: normal shaped bacteria; b: bacterial cells surrounded with CNPs; c: shrunken bacterial cell and loss of cell wall integrity; d: leakage of bacterial cell protein and release of intracellular components; e: loss of bacterial structure.

The antibacterial activity of CNPs against *A. hydrophila* subsp. *hydrophila* was demonstrated using TEM (Figure 10). The bacteria seemed to be in its typical shape (Figure 10a). CNPs were scattered around the bacterial cells (Figure 10b), adsorbed by cell wall, and altered cell wall permeability. Leakage of intracellular cytosolic components was also observed (Figure 10c-d). Bacterial cells started to lose architecture and died (Figure 10e).

#### 3.6.2. AgNps’ antibacterial activity via TEM

**Figure 11.**
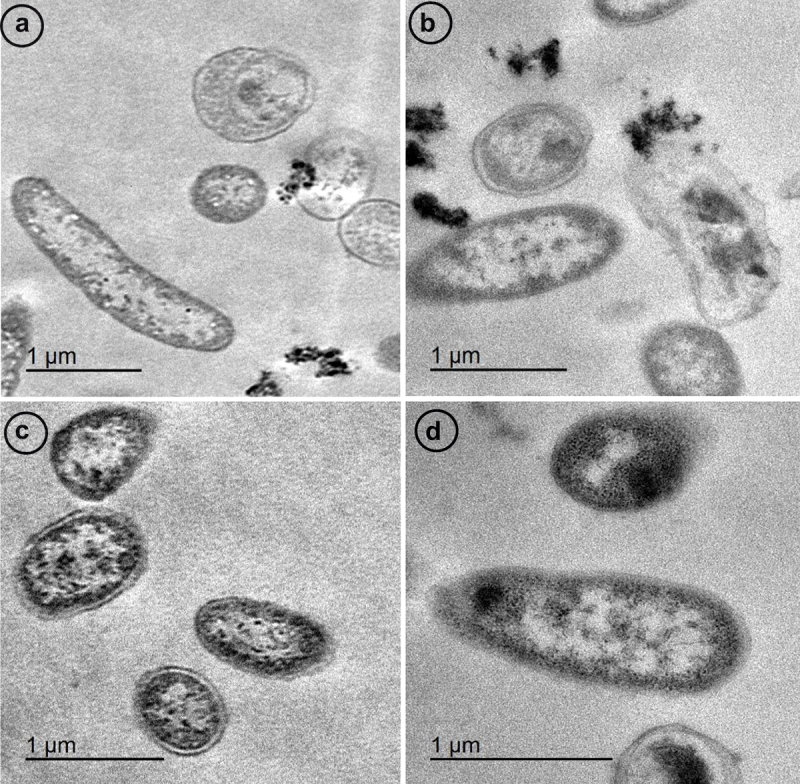
TEM images showed the microscopic alterations caused by AgNPs against *A.* *hydrophila subsp. hydrophila*; a: Bacterial cells without AgNPs; b: bacterial cells surrounded with AgNPs; c: AgNPs penetrated and entered inside the bacterial cell; d: loss of bacterial architecture.

AgNPs exhibited a better penetration ability to the bacterial cell wall. Infiltration of AgNPs to the bacterial cell could stimulate a direct reaction with lipids, proteins, and DNA. As a result, destabilization and apoptosis of bacterial cells were seen (Figure 11a,b,c,d).

### 3.7. In vivo studies against *A.* *hydrophila subsp. hydrophila* induced infection.

**Figure 12.**
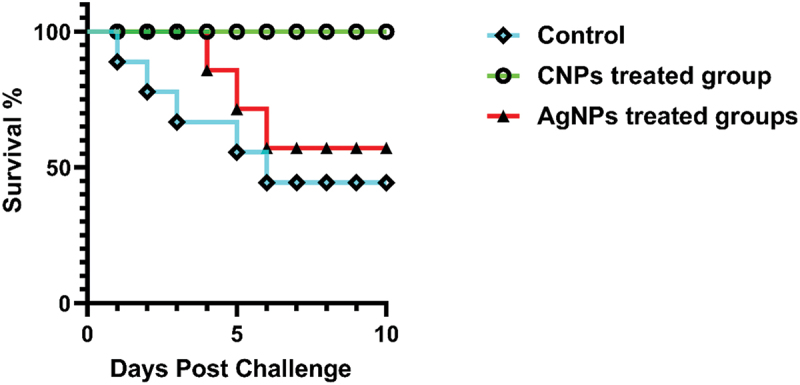
Survival plot of O. niloticus challenged with *A.* *hydrophila subsp. hydrophila* at a concentration of 3×10^7^ CFU/mL for the control, CNPs, and AgNPs treated groups, ten days post-challenge.

Incorporating CNPs into Nile tilapia diet for two months increased the survival percent and RLP to 100% with no mortalities recorded (*P* < 0.05), and improved resistance of treated fish against the induced *A. hydrophila* subsp. *hydrophila* infection. Moreover, AgNPs-treated groups showed a lower survivability and RLP reached 57.14%. The control non-treated group showed the lowest survival percent and RLP (Figure 12). Mild clinical signs were observed in CNPs- and AgNPs-treated groups 3-days post-challenge, including erratic swimming, lethargy, and hyperaemia at fin base. In contrast, fish fed the control diet showed severe clinical signs, such as ulcers, ascites, and external haemorrhage. Fish mortality was quickly reported in the control group 24-h post challenge.

## 4. Discussion

Nanoparticles have the potential to be applied as antibacterial agents in veterinary medicine and aquaculture [9]. Numerous fish pathogens, including Gram-positive and Gram-negative bacteria and fungi, were effectively inhibited by CNPs and Different preparations of AgNPs [11] [38,39].

The antimicrobial performance of nanoparticles is affected by their particle size and zeta potential [40]. In this study, the size distribution profile of CNPs constituted a typical quantity of nanoscale particles with an average size of 9.03 nm. The surface charge was positive and about+36.4 mV. The small particle size and the high positive surface charge of the particles make them more efficient and effective in penetrating and destroying bacterial cells.

This result was consistent with **Abdel-Razek et al** [10] who measured the size and charge of CNPs and investigated their antibacterial activity against several fish pathogens, the mean size was 35 nm and zeta potential was+61.2 mV. Furthermore, **Ali et al**. [41] synthesized CNPs with different sizes (95.7–112.4 nm) and positive surface charges (21.59–45.15) and demonstrated how the antimicrobial properties of CNPs can be enhanced by changing the size and surface charge of the prepared nanoparticles. Additionally, these findings concur with **Nikapitiya et al**. [42] who discovered that the average particle size distribution of synthetic CNPs was 181.2 nm and the zeta potential was+37.2 mV, respectively.

In our study, the distribution profile of AgNPs size represented a typical batch of nanoparticles with a range diameter of 10.6–15 nm. The surface charge of silver nanoparticles was about (−19.3) mV. In line with these findings, **El-Adawy et al**. [43] synthesized AgNPs with a narrow size distribution curve, the highest peak was recorded at 26.2 nm. The charge of the prepared particles was also negative (−17.1 ± 4.9 mV).

In addition, we determined the average particle size of CNPs and AgNPs using DLS analysis. The obtained result was significantly greater than that of TEM photography. This result might be caused by the hydrogel shell’s swelled state in an aqueous solution for DLS analysis, which had significantly shrunken in the dried state under TEM [42].

It is well known that substances with different charges attract and those with similar charges repel each other, so if we apply this principle to our study, the positive charge of CNPs in the present study may facilitate binding with the negatively charged bacterial cell membrane. CNPs cell wall binding could neutralize the surface charge of bacteria. Further interactions that denature membrane proteins and initiate phospholipid bilayer penetration, thereby increasing cell membrane permeability, may lead to cell membrane destabilization, intracellular chemical leakage, and ultimately cell death [10,44,45].

In the current study, diseased Nile tilapia that was clinically inspected, exhibited pale body surfaces, fin rot, exophthalmia, hyperaemia of the bases of the pectoral and ventral fins, and abdominal distension. The internal necropsy results of examined tilapia showed significant haemorrhagic patches in the liver, serous fluid accumulation in the gut, congestion in the liver and spleen, enlarged gall bladder filled with emerald-black fluid. These clinical signs and post mortem (PM) findings corroborate with earlier studies [8,21,46,47]. As a result of *A. hydrophila* invasion and colonization, small blood vessel rupture and subsequent extracellular component release can result in anaemia, ulceration, anorexia, and haemorrhagic sepsis [48].

In this study, 80 isolates were retrieved from the examined Nile tilapia. The recovered isolates were identified as *A. hydrophila* subsp. *hydrophila* with a percentage of 42.50%, *A. caviae* with a percentage of 38. 75%, and *A. punctata* with a percentage of 18.75%. Relatively similar findings were recorded by **Radu et al**. [49], who isolated 60 *Aeromonas* spp with a percentage of 69% from the examined retail fish samples in Malaysia and identified them as *A. hydrophila*, *A. caviae*, and *A. veronii* biovar *sobria.*

The most prevalent *Aeromonas* spp. in our study was *A. hydrophila* subsp. *hydrophila*. Comparable findings were obtained **by Tartor et al**. [47], revealing that the prevalence rate of *A. hydrophila* from Nile tilapia was 39%. Different fish species, geographic ranges, disease epidemiologies, and sample times could cause different prevalence rates.

These results were significantly concorded with **El-Bahar et al**. [8], who obtained *A. hydrophila* (22/80) representing 32.14% from diseased Nile tilapia^,^s total isolates. Moreover, in Uganda, **Wamala et al**. [50] identified several fish pathogens isolated from *O. niloticus* and *Clarias gariepinus* (African catfish) at a farm prevalence. The highest prevalence was recorded for *A. hydrophila* (43.8%). Five species of *Aeromonas* in Malaysia were isolated and identified. Among these isolates, *Aeromonas dhakensis* represented the most frequent pathogen (43%), followed by *Aeromonas veronii* (22%), *A. hydrophila* (20%), *A. caviae* (8%), and *Aeromonas jandaei* (7%) [51].

The highest prevalence of all retrieved isolates was in summer (41.25%) and spring (28.75%). This may be attributed to immunosuppression due to fluctuations in water temperature in the spring season or because of the stress due to high water temperature in the summer. Low dissolved oxygen also stresses the fish, weakens their immune response, and makes them more susceptible to bacterial infections. **Mzula et al**. [52] reported that the outbreaks of aeromonad diseases are seasonal based and commonly detected in the summer season between May and August.

In this study, all *Aeromonas* isolates tested positive for ciprofloxacin, ceftriaxone, streptomycin, and enrofloxacin. We discovered a significantly higher rate of resistance Against amoxycillin-clavulanic acid, followed by amoxycillin (AML) and oxytetracycline. These findings indicate the presence of virulent and MDR *Aeromonas* strains in cultured Nile tilapia. Misuse of these antimicrobials could explain the high rate of resistance observed in the isolated *Aeromonas* strains.

In agreement with earlier workers [5,47] who concluded that fluoroquinolones are almost universally effective against Aeromonads, most isolates in our study were sensitive to ciprofloxacin. This is supported by recent research in Egypt that all *A. hydrophila* isolated from mullet (*Mugil cephalus*) was sensitive to ciprofloxacin, a member of fluoroquinolones [53].

CNPs and AgNPs nanoparticles exhibited an *in vitro* inhibitory activity against the tested *A. hydrophila* subsp. *hydrophila*. The inhibition zones were 15 and 25 mm, respectively. This present result matched that previously observed by **Abdel-Razek** [10] who revealed that the growth of Gram-negative bacteria was inhibited due to the antibacterial activity of CNPs, and the inhibition zones were about (25–48 mm) in diameter. This may be due to two reasons, the first is the high negative charge on the Gram-negative cell surfaces, which makes the positively charged CNPs more adsorbable and of higher inhibitory effect against Gram-negative bacteria; the second is the lipopolysaccharide layer in the outer membrane of Gram-negative bacteria, which provides the bacterium with a hydrophilic surface. On the contrary, **Sayari et al**. [54] examined chitosan’s antibacterial impact against several bacterial pathogens, it was discovered that Gram-positive bacteria often exhibited more substantial and noticeable antimicrobial activities than Gram-negative bacteria. Chitosan’s inhibitory zones ranged from 10 to 35 mm. Similar results were reported by **Jeon et al** [55]. Our TEM images were captured to confirm the inhibitory activity of CNPs and AgNPs against *A. hydrophila* subsp. *hydrophila*. CNPs were adsorbed by the bacterial cell, causing destruction of the bacterial cell wall and irregularly condensed masses. The bacterial cells were disrupted to a certain degree with the leakage of cytosolic components. Additionally, AgNPs exhibited better penetration ability through the bacterial cell wall. AgNPs attach to the cell membrane, thus facilitating nanoparticle membrane attachment. Upon attachment, an evident morphological change was observed and can be characterized by cytoplasmic shrinkage and cell membrane rupture. Infiltration of Ag^+^ ions into the bacterial cell could stimulate a direct reaction with lipids, proteins, and DNA. As a result, destabilization and apoptosis of bacterial cells may occur. In this regard, the mechanism of antibacterial activity of AgNPs was described in the study of **Singh et al**. [56] first, the nanoparticles adhere to the bacterial cell membrane, then they can penetrate it and enter the cell. This impairs crucial cell functions and results in cell apoptosis and death. **Sat et al**. [57] explained that mechanism of apoptosis in *Escherichia coli* bacteria was supported by two factors: an irregular decrease in cell size and DNA fragmentation. In addition, **Bortner and Cidlowski** [58] described that DNA fragmentation is the final step in apoptosis that proceeds with the reduced cell size. This stage of bacterial cells is called shrunken cells or apoptotic bodies.

Similar findings were declared by **Divya et al** [59]. who attributed the bactericidal action of CNPs to the amount of intracellular material and nucleic acid released from the challenged bacterial cells due to cell membrane leakage and loss of bacterial architecture and cell wall integrity. Moreover, AgNPs Interact with bacterial cell ribosomes cause ribosomal denaturation, inhibition of protein synthesis, and a severe damage of the bacterial cells [60].

Standard TEM preparation techniques generally require the complete removal of the suspending liquid in the samples to be examined. Drying often introduces artefacts, which can obscure the state of the dispersion prior to drying and can cause artefacts on the examined specimens. A simple protocol for preventing drying artefacts and preserving in situ colloidal features of nanoparticle-bacterial suspension during sample preparation was used to address this issue [61].

Another TEM artefact is the formation of aggregates. The preparation process of any sample typically consists of drop-casting and drying particle dispersion on a TEM grid. This process usually results in the formation of nanoparticle aggregates [62,63]. We followed the approach of **Michen et al**. [61] that depends on preventing particle aggregation, minimizing dewetting and maintaining hydration.

Incorporating CNPs and AgNPs in the tilapia diet potentially increased the RLP and survival rate of the challenged fish groups compared with the control. These results have been echoed more recently by **Nikapitiya et al**. [42] who demonstrated a higher survival rate against pathogenic *A. hydrophila* in zebrafish larvae exposed to CNPs (5 μg/mL) at 5 days post fertilization (5 dpf) stage. Due to its potent immune modulatory properties, CNPs exposure at 5 μg/mL may also improve the immune response and lead to disease resistance against *A. hydrophila*. **Saleh et al**. [64] assessed the effect of CNPs on the relative percent of survival (RPS) of rainbow trout, *Oncorhynchus mykiss* fed CNPs mixed diet for 21 days. After a challenge experiment lasted for 28 days, the RPS of CNPs treated fish groups was 80% when compared to the control groups. Diet supplementation with CNPs had beneficial effects in protecting Nile tilapia from several bacterial pathogens [10]. Researchers had detected that CNP-fed fish differed in their resistance to various aquatic bacterial pathogens. These differences could be attributed to changes in the permeability of each bacterium’s cell wall, resulting in internal osmotic imbalances and, as a result, inhibiting the bacterial growth [65].

## 5. Conclusion

In brief, our findings revealed the presence of virulent and multi-drug resistant *Aeromonas* strains as an implication of antibiotics misuse in Nile tilapia culture. We focused on the study of antibacterial effect of CNPs and AgNPs as possible alternatives to antibiotics. We concluded that CNPs and AgNPs both have *in vitro* and *in vivo* antibacterial inhibitory effect against *A. hydrophila* subsp. *hydrophila* affecting Nile tilapia. Further studies on the antimicrobial effect of CNPs and AgNPs against other fish pathogens is required to establish them as effective broad-spectrum antimicrobial supplements in controlling aquaculture threatening pathogens.
